# Animal mesenchymal stem cell research in cartilage regenerative medicine – a review

**DOI:** 10.1080/01652176.2019.1643051

**Published:** 2019-08-28

**Authors:** Mudasir Bashir Gugjoo, Amarpal, Mujeeb-Ur Rehman Fazili, Mohmmad Abrar Gayas, Raja Aijaz Ahmad, Kuldeep Dhama

**Affiliations:** a Division of Veterinary Clinical Complex, FVSc and AH, SKUAST, Srinagar, India;; b Division of Surgery, Indian Veterinary Research Institute, Bareilly, India;; c Division of Surgery and Radiology, FVSc and AH, SKUAST, Srinagar, India;; d Division of Pathology, Indian Veterinary Research Institute, Bareilly, India

**Keywords:** Animals, cartilage regenerative medicine, chondrogenesis, clinical studies, mesenchymal stem cell, preclinical studies

## Abstract

Healing of articular cartilage is a major clinical challenge as it also lacks a direct vasculature and nerves, and carries a limited number of resident chondrocytes that do not proliferate easily. Damaged articular cartilages are usually replaced by fibrocartilages, which are mechanically and structurally weaker and less resilient. Regenerative medicine involving stem cells is considered to have a definitive potential to overcome the limitations associated with the currently available surgical methods of cartilage repair. Among various stem cell types, mesenchymal stem cells (MSCs) are preferred for clinical applications. These cells can be readily derived from various sources and have the ability to trans-differentiate into various tissue-specific cells, including those of the cartilage by the process of chondrogenesis. Compared to embryonic or induced pluripotent stem cells (iPSCs), no ethical or teratogenic issues are associated with MSCs. These stem cells are being extensively evaluated for the treatment of joint affections and the results appear promising. Unlike human medicine, in veterinary medicine, the literature on stem cell research for cartilage regeneration is limited. This review, therefore, aims to comprehensively discuss the available literature and pinpoint the achievements and limitations associated with the use of MSCs for articular cartilage repair in animal species.

## 1. Introduction

### 1.1. Cartilage structure and its lack of regenerative potential

The articular cartilage is an opalescent layer of hyaline tissue that furnishes an exceptional resilience and almost frictionless movement to the diarthrodial joints (Mankin 1984). Cartilage is a highly differentiated tissue maintained by a single exiguously distributed cell type, known as the chondrocyte, and is devoid of direct blood vessels, lymphatics or nerves (Kinner et al. 2005; Duarte Campos et al. 2012). Like other tissues, stem cells are also present in the articular cartilage but their role remains to be elucidated (Williams et al. 2010; Pretzel et al. 2011; Nelson et al. 2014). Overall, the cartilage structure is the same in all species and comprises of superficial, radial, and deep zones. The deep zone is separated from the subchondral bone by a wavy tidemark zone. The cartilage matrix mainly comprises water, collagen (imparts tensile strength), and proteoglycans (provide functional resistance to compression) (Maroudas 1979; Pool 2001). The thickness of the articular cartilage varies from one type of joint to another and also with the age of the animal (Athanasiou et al. 1995). The average thickness of the knee cartilage in adult rabbits, sheep, dogs, goats, horses, and humans is 0.3, 0.4–0.5, 0.6–1.3, 0.7–1.5, 1.5–2.0, and 2.2–2.5, respectively (Frisbie et al. 2006).

The articular cartilage has limited healing potential because it is a terminally differentiated tissue lacking a direct connection with the vasculature and innervations. Osteoarthritis (OA), a common cause of joint dysfunction, may be induced either by trauma or auto-immune reactions. Trauma-induced defects in the cartilage may be either of the partial- or full-thickness type. Partial-thickness defects are confined to the cartilage tissue itself while those of full-thickness penetrate the subchondral bone (Hunziker 1999; Gugjoo et al. 2016). Due to absence of the fibrin clot and thus, reparative stem cells, partial-thickness defects do not heal spontaneously. These defects are analogous to fissures or clefts seen in the early stages of OA (Hunziker 1999). Although the full-thickness defects heal spontaneously, they result in a mechanically and structurally weakened fibrous tissue that lacks integration with the native cartilage (Hunziker 1999; Arican et al. 2006; Tiwary et al. 2014).

Auto-immune diseases like rheumatoid arthritis involve a more generalized affection of the joints with progressive cartilage erosion. OA affects about 21.4 and 20.0% of the human (Barbour et al. 2016) and dog (Johnston 1997) population, respectively. In horses, OA is one of the most common causes of lameness. A survey has reported that approximately 33% of the equine patients carry cartilage lesions associated with OA (Rose 1977). As the cartilage is a weight-bearing tissue, its erosion from joints elicits pain and progresses to the loss of joint function. Therefore, it is imperative to develop therapeutic approaches that can regenerate the integrated hyaline tissues for better joint rehabilitation (Hunziker 1999; Gugjoo et al. 2016; Juneau et al. 2016).

### 1.2. Why mesenchymal stem cell therapy?

Majority of the current treatment options available for cartilage rehabilitation fail to regenerate the cartilage structure. Surgical procedures like induction of microfractures, subchondral bone drilling, lavage and debridement, perichondral arthroplasty, periosteal arthroplasty, autologous osteochondral transplantation, and autogenetic cancellous bone grafts have failed to regenerate the articular cartilage effectively (Tiwary et al. 2014; Gugjoo et al. 2016; Jeuken et al. 2016; Gugjoo et al. 2017; Wang 2017). To address this issue, there is an increasing focus on the study of cartilage in the field of regenerative medicine and different ways of employing various components, including the cells for cartilage regeneration being devised (Kaiser 1992; Ehnert et al. 2009). The cells employed for this purpose are either stem cells or tissue-specific chondrocytes. Chondrocytes are primarily employed for majority of the cellular therapies (approximately 80%) in cartilage regenerative medicine (Fraser et al. 2006). Results of the chondrocyte implantation (ACI) technique are appreciable but its clinical applications are limited due to limited availability of their sources, likelihood of the cells to dedifferentiate into fibroblasts, and degeneration in the pre-damaged cartilage (Punwar and Khan 2011). Additionally, the aging chondrocytes show declining mitotic and synthetic activity and synthesize smaller and less uniform aggrecan molecules bearing less functional link proteins (Adkisson et al. 2001).

Comparatively, although stem cells contribute to only about 15% of the cellular therapies for cartilage regeneration, their involvement is increasing with each passing day (Fraser et al. 2006). Stem cells harvested from numerous sources have the ability to differentiate into different lineages based on the available niche. It is considered to be an all-in-one solution for diverse ailments including those of the cartilage. Among various types of stem cells, the adult multi-potent MSCs mainly contribute to regenerative therapeutics. These cells are readily available from numerous sources, easily harvested and have an ability to differentiate into mesodermal and extra-mesodermal tissues. Furthermore, the teratogenic and ethical issues associated with embryonic stem cell (ESC) and induced pluripotent stem cells (iPSCs) therapy are not encountered with the application of MSCs (Cardoso et al. 2017; Wang et al. 2017; Gugjoo, Amarpal, Sharma, et al. 2019).

**Figure 1.**
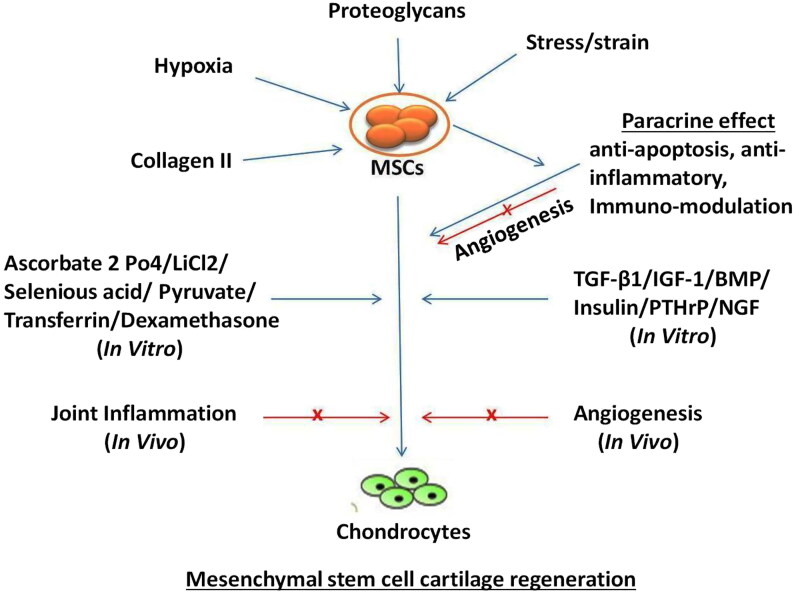
*In vitro* and *in vivo* mesenchymal stem cell cartilage regeneration. Blue arrows represent facilitation of chondrogenesis; red arrows represent inhibition of chondrogenesis; x represents blocking the pathway.

Extensive literature available on the use of MSCs have variably supported their therapeutic potential (Carrade, Affolter, et al. 2011; Carrade, Lame, et al. 2011; Spaas, Oosterlinck, et al. 2012; Spaas, Guest, et al. 2012 ; Iacono et al. 2016; Gugjoo et al. 2017; Kazemi et al. 2017; Kriston-Pál et al. 2017; Feng et al. 2018; Zhang et al. 2018). Terminal differentiation or paracrine action of the MSCs can provide relevant clinical benefits. Initially, it was considered that the MSCs contribute to lesion healing by integrating directly into the tissue. However, this mechanism is considered relatively insignificant compared to their trophic effect (Stewart and Stewart 2011; Gugjoo, Amarpal, Makhdoomi et al. 2019). The trophic action involves the release of a diverse array of cytokines, growth factors, chemokines, and immuno-modulatory proteins (Stewart and Stewart 2011; Gugjoo, Amarpal, Fazili, et al. 2019). This action may be induced by the secretion of proteins or peptides and hormones, transfer of mitochondria through tunneling nanotubes or microvesicles, and/or the transfer of exosomes or microvesicles containing RNA and other molecules (Figure 1) (Spees et al. 2016; Gugjoo, Amarpal, Makhdoomi, et al. 2019).

The characteristic immuno-compromised, immuno-modulatory, and anti-inflammatory features of MSCs make them more appropriate therapeutic agents for OA. The MSCs express MHC-I and variably express MHC-II or T-cell co-stimulatory molecules, particularly those derived from for instance the equine bone marrow (BM), umbilical cord (UC) matrix, and/or UC blood (De Schauwer et al. 2014; Schnabel et al. 2014; Berglund et al. 2017). Equine MSCs express cytokines such as TSG-6 (receptor antagonist of pro-inflammatory cytokine IL-1) and IL1-Ra (anti-inflammatory action and inhibitor of matrix metalloproteinases) that reduce inflammation (Kode et al. 2009). MSCs obtained from different sources decrease lymphocyte proliferation, produce tumor necrosis factor-α (TNF-α) and interferon-γ (IFN-γ), and increase the secretion of prostaglandin (PGE2) and interleukin-6 (IL-6) (Kang et al. 2008; Kode et al. 2009; Peroni and Borjesson 2011; Carrade et al. 2012; Colbath et al. 2017; Yang et al. 2018). Despite these desirable features observed *in vitro*, these cells cannot be used as definitive therapy in clinical conditions as contrary to their laboratory observations, under *in vivo* conditions, these cells are uncontrolled. Moreover, these cells do not remain confined to the implantation site but migrate and reach other sites (migration and homing) (Guest et al. 2008; Kode et al. 2009; Stewart & Stewart 2011). Therefore, the results obtained in *in vitro* studies may not be reproduced exactly as those under *in vivo* conditions.

The literature cited in this manuscript has been retrieved from various authentic sources such as MEDLINE, PubMed, PubMed Central, and ScienceDirect. Initially, data on general studies relevant to animal MSCs were collected. From the collected material, the literature on chondrogenic studies was sorted. Application of the collected information was aimed at evaluating the chondrogenic potential of MSCs of animal origin both *in vitro* as well as *in vivo*. Additionally, our own experience regarding this topic has been shared.

## *2. In vitro* studies on mesenchymal stem cell

MSCs are present in majority of the tissues of an adult individual and are characterized by specialized properties such as self-renewal, multiplication, immuno-modulation, and multi-lineage differentiation (Gugjoo, Amarpal, Makhdoomi, et al. 2019). Isolation followed by *in vitro* culturing of the stem cells is imperative because they are present in limited concentrations in the tissues. MSCs usually have a limited expansion potential and after a certain number of passages, the cells tend to become senescent as their viabilities and proliferation potentials decrease. Such variability is associated with the tissue source, age of the animal, and techniques employed for their culturing (Corradetti et al. 2013; Xiong et al. 2014). Many variations have also been reported among different breeds. As an illustration, BM-MSCs from the German Shepherd, Labrador, and Golden Retriever dogs tend to undergo senescence rapidly compared to those derived from the Border Collie, Malinois, and Hovawart breeds (Bertolo et al. 2015).

To confirm the presence of MSCs, *in vitro* cellular and molecular characterization is performed based on the recommendations of the International Society for Cell Therapy (ISCT), which include evaluation of plastic adherence, expression of surface receptors (CD105, CD90, CD73, and CD90) and inability to express CD45, CD34, CD14 or CD11b, CD79a or CD19, and HLA-DR molecules. Furthermore, the cells should at least differentiate into the osteogenic, chondrogenic and adipogenic lineages in their respective media (Dominici et al. 2006). Although these recommendations were earlier applicable only for the human MSCs, the same recommendations have also been adopted for the characterization of animal MSCs (De Schauwer et al. 2011; Pascucci et al. 2011; Gugjoo et al. 2015; Hillmann et al. 2016; Broeckx et al. 2019). MSCs generally meet the criteria for plastic adherence and pluripotency, but fail to meet those on surface marker expression. The differences in the expression patterns of their surface markers vary with the type of antibody used, cell sources, and methods employed for culturing (Ranera et al. 2011; Screven et al. 2014).

### 2.1. In vitro culturing of MSCs for chondrogenesis

*In vitro* chondrogenesis may be achieved either in 2D or 3D culture systems. However, the efficiency of chondrogenesis tends to be lower in the former system. An *in vitro* scaffold-based 3D culture system for chondrogenesis is being increasingly studied compared to the commonly studied scaffold-free one. Such a system tends to support cell aggregation, mimic the *in vivo* environment, improve cell communication, and produce the extracellular matrix (ECM) (Liu et al. 2016; Nam et al. 2018). The scaffold-free 3D cultures which are commonly studied include pellet and micromass culture systems. In the pellet system, cells in the pellet form are entrapped into the secreted ECM, unlike that of the micromass culture. The two systems are variably supported for chondrogenic studies. In general, the efficiency of chondrogenesis is enhanced in the micromass culture technique but the pellet culture is considered to be more useful for clinical applications. This preference to the pellet culture system is due to its enhanced efficiency in generating sufficient chondrocytes compared to that of the micromass culture technique (Nam et al. 2018). Some *in vitro* studies have demonstrated that the cartilage may be generated by recapitulating various developmental processes of mesenchymal condensation. The cartilage tissue thus secreted may resemble a hyaline tissue, both, in physiological stratification as well as biomechanical features (Nam et al. 2018). For instance, evaluation of the phenotype of chondrogenically differentiated equine BM-MSCs reveals the presence of primary cilia and an intense synthetic and metabolic activity comparable with that of the chondrocyte phenotype, depicting a steady transformation of MSCs into the actual chondrogenic lineage (Luesma et al. 2016). Overall, native chondrocytes are considered superior in their ability to secrete a matrix with better mechanical properties compared to the hydrogel laden BM-MSCs (Mauck et al. 2006).

The initial step involved in the *in vitro* chondrogenesis of MSCs is the creation of condensed mesenchymal cell bodies (CMBs). These consist of packed MSCs that have increased cell–cell contact but do not undergo any proliferation. This is followed by the process of chondrogenic differentiation utilizing different growth factors (Hall and Miyake 2000; DeLise et al. 2000; Vickers et al. 2010; Bhumiratana et al. 2014). Under *in vitro* conditions, the CMBs generate tissues comparable to those of the native cartilages on osseous surfaces and also develop mechanically strong, completely integrated interfaces between the cartilages and their tissues (Bhumiratana et al. 2014). Osteogenic differentiation of MSCs primarily takes place by the intra-membranous ossification pathway (Scotti et al. 2010). To create an environment favorable for chondrogenesis, it is imperative to push MSCs down the endochondral ossification pathways involving condensation of MSCs followed by chondrogenic differentiation and formation of the cartilage template, following which, their progress may be restricted before the osteogenic pathways ensue (Kozhemyakina et al. 2015). The chondrogenesis is supported by the expression of *Sox9* which represses chondrocyte hypertrophy possibly by inhibiting Runx2 (Zhou et al. 2006), *Wnt* (Topol et al. 2009), *Col10a1*, and VEGFA (Hattori et al. 2010; Leung et al. 2011), required for osteogenesis. The upregulation of *Sox6* that in turn promotes the *Sox9* gene may be promoted by pyruvate dehydrogenase kinase isoform 2 (PDK2) (Wang et al. 2017).

In a previous study, evaluation of the chondrogenic potential of the MSCs was accomplished by the incorporation of dexamethasone and TGF-β1 into the culture (Johnstone et al. 1998). Subsequent successful chondrogenic studies utilized many other growth factors and chemicals including the insulin-like growth factor-1 (IGF1), bone morphogenetic proteins (BMPs), parathyroid hormone-related peptides (PTHrP), insulin, ascorbate-2-phosphate, selenious acid, transferrin, sodium pyruvate, nerve growth factor (NGF), and lithium chloride (Johnstone et al. 1998; Mackay et al. 1998; Yoo et al. 1998; Lee et al. 2000; Sekiya et al. 2001; Zhang et al. 2004; Sekiya et al. 2005; Kim et al. 2008; Pei et al. 2008; Guilak et al. 2010; Lu et al. 2017). Different growth factors used at varying concentrations result in different expressions of these cells. After 14 d of cell culture, TGF-β1 (10 ng/mL) (Zeiter et al. 2009) and TGF-β3 (1, 10, and 100 ng/mL) (Goldman and Barabino 2016) induced the expression of chondrogenic genes in bovine BM-MSCs. Contrarily, the cells may or may not have been affected by BMP-2. A study reported that BMP-2 (50 ng/mL) may not have any effect on the chondrogenesis of bovine MSCs (Zeiter et al. 2009) while in other studies, it was reported that BMP-2 (1, 10, and 100 ng/mL) promotes the differentiation of these cells toward the osteogenic lineage (Goldman and Barabino 2016). A combination of growth factors (BMP-2 + TGF-β_1_) can also differentiate the MSCs toward the chondrogenic lineage (Branly et al. 2017). This variability in results depicts the role of other culture factors in the differentiation of the MSCs. Growth factors tend to induce differentiation of the MSCs toward a hypertrophic chondrocyte template (type X collagen synthesis) that results in their divergence toward osteogenesis instead of restricting them into following the pathway for chondrogenesis (Gugjoo et al. 2016).

Recently, new techniques are being studied to evaluate anti-angiogenesis of the MSCs transfected with a non-viral endostatin plasmid. This is aimed at regenerating avascular tissues like the cartilage (Sun et al. 2009). Blockade of the vascular endothelial growth factor, one of the significant contributors in the development of osteophytes in OA, prevents chondrocyte hypertrophy of the MSCs in a lab animal model (Matsumoto et al. 2009), and subsequently, prevents progression of the disease. However, such an inhibition does not negatively affect either the viability of the cells or their chondrogenesis (Jeng et al. 2010). Thus, further studies are required to develop techniques that restrict the MSCs to chondrogenesis without progression to osteogenesis.

### 2.2. Effect of mechanical factors on the in vitro chondrogenesis of MSCs

Mechanical factors may or may not affect the differentiation of MSCs. No effect on the differentiation of bovine MSCs was observed when subjected to hydraulic pressure (0.5–3 MPa for 4 h/d) (Zeiter et al. 2009), whereas shear stress (10 Dyn/cm^2^) induced the differentiation of these cells toward the chondrogenic or osteogenic lineages (Goldman and Barabino 2016). Adverse effects of inflammatory mediators like IL-1β on the chondrogenesis of bovine MSCs may be prevented by combining the mechanical (electromagnetic fields) and growth factors (TGF-β3) (Ongaro et al. 2015). However, to restrict cells to a specific chondrogenic lineage and prevent the induction of a hypertrophic osteogenic pathway, shear stress may be employed along with a sufficient concentration of TGF-β3 (Goldman and Barabino 2016). Therefore, mechanical loading of the bovine BM-MSCs by rapid chromatin condensation may induce their differentiation toward the chondrogenic lineage. This may be coincident with the upregulation of fibrochondrogenic phenotype marker expression (Heo et al. 2015). An extracorporeal shockwave therapy used for promoting osteogenesis reduces chondrogenesis in the 3-D cultures of BM-MSCs, possibly through the regulation of adenosine release and activation of the A2B receptor (Tan et al. 2017).

Hypoxia, which is normally present in the cartilage environment, induces chondrogenesis of human and sheep MSCs (Zscharnack et al. 2009; Guilak et al. 2010; Ronzière et al. 2010; Bornes et al. 2015). Although hypoxia (5%) lowers the proliferation potential of adipose-derived human MSCs (AD-MSCs), it causes a three-fold increase in the secretion of collagen and proteoglycans (Guilak et al. 2010). At lower oxygen concentrations (2%), the MSCs get arrested during the process of chondrogenesis. They do not undergo hypertrophic maturation despite addition of BMP-1 and BMP-2 (Ronzière et al. 2010). Significantly superior expressions of aggrecan and collagen II mRNAs, GAG quantities and proteoglycan staining are observed in BM-MSCs seeded with collagen and hyaluronic acid under hypoxic conditions when compared to cells in which normoxia is maintained. However, it also tends to increase hypertrophic chondrogenesis that may progress toward osteogenesis (Bornes et al. 2015). Thus, further research is required to study the successful regeneration of the hyaline cartilage without its progression toward the fibrocartilage phenotype and/or osteogenic lineage.

### 2.3. Effect of native environmental factors

Apart from various exogenous growth factors, numerous *in vitro* factors that resemble the *in vivo* microenvironment may favor chondrogenesis. The cartilage-derived ECM favors the chondrogenic differentiation of rat BM stromal cells without the influence of any exogenous growth factor (Yin et al. 2016). Addition of laminarin (beta-(1→3)-D-glucan) reduces the proliferation and chondrogenic differentiation of the MSCs derived from the rat BM-MSCs (Larguech et al. 2017). The addition/replacement of different substrates to the culture media may lead to preferential chondrogenesis. Compared to the cultures maintained under conditions of normoxia, the hypoxic cultures of sheep BM-MSCs seeded with collagen and hyaluronic acid demonstrate superior expressions of aggrecan and collagen II mRNAs, amount of glycosaminoglycans, and proteoglycan staining (Bornes et al. 2015). Variable concentrations (25, 50, and 100%) of synovial fluid support the viability, proliferation, and chondrogenic differentiation of equine BM-MSCs (Boone et al. 2018). When goat BM-MSCs (encapsulated in polyethylene glycol) are cultured in collagen (Collagen I and II)-based and hyaluronic-based extracellular matrices, they are directed to the chondrogenic and osteogenic lineages, respectively (Hwang et al. 2011). 3D cultured synthetic biodegradable scaffolds also direct goat BM-MSCs to the chondrogenic lineage. Chondroitin sulfate promotes the mesenchymal condensation of goat MSCs and thereby upregulates their cartilage-specific genes. Additionally, supplementation of polyethylene glycol to chondroitin sulfate may prevent hypertrophic chondrocyte formation (Varghese et al. 2008). Sheep MSCs, under *in vitro* conditions (placed on porous calcium polyphosphate along with tri-iodothyronine), secrete cells of the osteochondral tissue. The MSCs tend to form a cartilaginous structure together with the osteogenic tissue. It was found that the osteogenic tissue remained over the calcium polyphosphate, over which, the cartilaginous tissue was formed (Lee et al. 2015). Thus, the microenvironment plays a significant role that enables the cells to exhibit their characteristic properties, to which currently very limited exogenous control can be applied under *in vivo* conditions.

### 2.4. Effect of cell source on the in vitro chondrogenesis by MSCs

The cell source may also affect musculoskeletal differentiation, including chondrogenesis (Gugjoo et al. 2017; Gugjoo et al. 2018). Among various cell sources, the BM and adipose tissue (AD)-derived MSCs are commonly utilized for the *in vivo* clinical studies and trials (Nam et al. 2018). However, cells derived from different sources may have variable differentiation potentials. MSCs derived from the synovium (S-MSCs) enable the formation of a large and heavy cartilage pellet compared to the BM-MSCs, AD-MSCs, periosteal-MSCs (P-MSCs), and muscle-MSCs (M-MSCs) (Shirasawa et al. 2006). The cell concentrations of equine BM are lesser compared to those of the AD tissue (Toupadakis et al. 2010). However, the equine BM-derived cells demonstrate superior activity for differentiation into musculoskeletal tissues (Kisiday et al. 2008; Vidal et al. 2008). Stem cells derived from the equine BM, AD, and tendons proliferate faster than the UC-MSCs under *in vitro* conditions (Burk et al. 2013; Barberini et al. 2014). It was reported that 80% confluency of BM-MSCs, AD-MSCs, and UC-MSCs was achieved in 11 d, 7.3 ± 1.52 and 15.25 ± 6.65 d, respectively (Barberini et al. 2014). The growth rate of UC-MSCs, however, increases upon addition of higher concentrations (20%) of fetal bovine serum (Toupadakis et al. 2010). The chondrogenic potential of equine BM-MSCs is higher than that of the AD-MSCs (Vidal et al. 2008). Sheep perivascular stem cells (considered as natural ancestors of the MSCs) may have better chondrogenic potential as compared to that of sheep BM-MSCs, as evidenced by increased synthesis of the ECM (Hindle et al. 2016). In one of the studies, it was found that the chondrogenic potential of ovine UC-MSCs was superior to that of the BM-MSCs (Burk et al. 2013). MSCs from fetal membranes are generally considered fast growing with better differentiation properties (Somal et al. 2016). The characteristics of BM-MSCs harvested from the equine sternum and ileum were comparable (Lombana et al. 2015). Contrarily, AD-MSCs of the intra-articular fat pad showed superior chondrogenic potential compared to the non-articular fat-derived MSCs (Stewart 2011). This indicates that the cells harvested from different regions of the body but of same individual may or may not demonstrate similar characteristics. Thus, while instituting the MSC therapy, it is imperative to consider characteristics of the cells based on their source.

### 2.5. Effect of donor age, health status, and breed on in vitro chondrogenesis of MSCs

The cellular characteristics may also vary with age and health status of the donor. MSCs undergo age-related functional loss with respect to their differentiation potentials and proliferation capacities (Kretlow et al. 2008; Huang et al. 2010; Yu et al. 2011; Peffers et al. 2016). This has implications on the healing potential and health status of the individuals as degeneration of the tissues ensues subsequently with aging (Zaim et al. 2012). A reduction in the potentials for proliferation (40%) and osteogenesis is observed in BM-MSCs of aged dogs (Volk et al. 2012). A higher level of population doubling and expression profile of surface markers (CD73 and CD80) and pluripotency markers (Oct3/4 and Nanog) were observed in the MSCs derived from young dogs compared to those harvested from aged dogs (Lee et al. 2017). Therefore, to obtain better results, young donors may preferentially be selected to harvest MSCs for orthopedic applications. Comparison of equine MSCs derived from the synovial fluid and synovial membranes of diseased joints (osteoarthritic and osteochondrosis dissecans) with those of healthy tissues has shown similar phenotypic and multipotency potentials but the chondrogenic potential of MSCs harvested from healthy tissues is better than those of the former (Fülber et al. 2016). Furthermore, studies on the pathophysiological conditions in different species and their influence on the MSCs are needed to understand the correct approach for employing the cells for our benefit. Additionally, characteristics of the BM-MSCs may vary depending on the breed as well. From the standpoint of chondrogenic differentiation, the BM-MSCs derived from the Labrador, Retriever, and Hovawart dog breeds demonstrated better chondrogenesis compared to those of the Border collie and German shepherd breeds (Bertolo et al. 2015). All these complicacies and variabilities in the features of MSCs pose impediments in the determination of a definitive stem cell therapy.

MSCs, as discussed above, are variably affected by donors, tissue sources, and may even vary within given cell populations. Such variabilities complicate their use in regenerative medicine. As detailed above, these conventional assays are usually applied to measure properties of the MSCs en masse, and hence, fail to control a particular cell population. Extensive variability within clonal MSC populations also exists. This affects their functional differentiation capacities, molecular state biophysical properties, and paracrine effects (McLeod and Mauck 2017). Recently, a study on mice showed that the transcriptomic profile and chromatin accessibility signatures may impart such differences. It was also demonstrated that chromatin accessibility signatures may be more accurate than those of the transcriptomic profiles. The transcription factors associated with the manifestation of these characteristic differences of the cells depending on their sources have been characterized (Ho et al. 2018). Thus, a system needs to be identified that could locate them individually for appropriate clinical applications in tissue engineering and regenerative medicine (McLeod and Mauck 2017).

## *3. In vivo* preclinical experimental models/clinical studies

Numerous *in vivo* chondrogenic studies involving MSCs have been conducted on almost all the veterinary relevant mammalian species like sheep, goat, dog, and horse, with the exception of bovines, cats, and swine. In the majority of these studies, implantation of the MSCs has yielded good results. However, the majority of the studies were non-uniform with differences in the sources of cells, culture techniques, dosages, passage numbers, implantation methods, growth factors, and type of scaffolds. Mostly, cells derived from either allogeneic or autologous transplants have been used in these studies. Allogeneic MSCs implanted either once or repeatedly have been reported as safe and are not known to induce any hypersensitivity reactions (Vangsness et al. 2014; Vega et al. 2015; Ardanaz et al. 2016). It has been reported that these cells survive even up to 14 weeks after *in vivo* implantation (Feng et al. 2018). Apart from the allogeneic cells, human xenogenic stem cells too are known to give better results compared to those of the control. This may be attributed to their characteristic immunocompromised feature. However, the use of xenogenic MSCs is currently not recommended in clinical trials. MSCs have anti-inflammatory properties but high-end inflammation usually reduces their ability for chondrogenic differentiation without affecting their phenotypic characteristics and proliferation potential (Ando et al. 2012; Zayed et al. 2016). In equine OA, the limited efficacy of their MSCs may be explained by an increased expression of the adhesion molecule, a decrease in the migration of related genes (Barrachina et al. 2016; Reesink et al. 2017), and the production of glycosaminoglycans (Zayed et al. 2016). Pro-inflammatory cytokines like IL-1β, IL-17, and TNF-α decrease the expression levels of cartilage-specific genes like *SOX-9* and *TGF-β1*, and those encoding aggrecan, collagen II (Kondo et al. 2013; Zayed et al. 2016), and galactin (Reesink et al. 2017) in the MSCs. However, the expression levels in MSCs may decrease depending upon the type of their source. In the presence of inflammatory mediators, a reduction in the expression of aggrecan only is seen in the MSCs derived from the synovial fluid when compared to that of the BM-MSCs (Zayed et al. 2016). The role of inflammation on the pathways related to the stem cell growth and cytokine expression should be further explored to have a better understanding of the limitations in the efficacy of stem cell therapy during inflammatory conditions. Currently, the initial step in cartilage rehabilitation should be aimed at decreasing the inflammation by the adoption of anti-inflammatory drugs. This may be followed by the application of MSCs for better results. The *in vivo* articular studies conducted in different animals are described below.

### 3.1. MSC studies in sheep

Numerous studies on the repair of osteochondral ailments using MSCs have been conducted in sheep. Most of the studies (listed in Table 1) have reported the usefulness of MSCs for improving the condition of joint ailments compared to that of the control (Guo et al. 2004; Feitosa et al. 2010; Zscharnack et al. 2010; Al Faqeh et al. 2012; Caminal, Moll, et al. 2014; Caminal, Fonseca, et al. 2014; Song et al. 2014; Garcia et al. 2014; Zorzi et al. 2015; Desando et al. 2016; Whitehouse et al. 2017; Abdalmula et al. 2017; Feng et al. 2018). In addition to cultured MSCs, the BM aspirate too has been reported to improve cartilage healing (Duygulu et al. 2012). However, no obvious improvement compared to the control was observed in a single study upon use of the BM aspirate (Delling, Brehm, Metzger, et al. 2015). MSCs implanted into the joints remain viable and attach themselves to the joint structures (Delling, Brehm, Ludewig, et al. 2015; Feng et al. 2018).

**Table 1.** Chondrogenic *in vivo* preclinical experimental mesenchymal stem cell studies in sheep.

Model type	Number of animals	Model defect size/study period	Biomaterial used	Cell dose	Evaluation criteria	Overall result	References
Medial femoral condyle defect	28 (*n* = 16 cell along β-TCP with treated; *n* = 8 in β-TCP only and *n* = 4 in control)	8 mm (diameter) and 4 mm (depth)/24 weeks	Autologous BM-MSCs + beta-tricalcium phosphate (β-TCP)	3 × 10^7^	Macroscopic observation, histological, immuno-histochemical, biochemical analysis	Experimental animal defects were resurfaced with hyaline-like tissue. An ideal interface formed between the engineered cartilage, adjacent normal cartilage, and the underlying bone	Guo et al. (2004)
Osteonecrosis of femoral head	8 animals (4 in control group; 2 each in sheep MSC group and human MSC group treated after 8 weeks of induced necrosis)	10 mL of absolute ethanol induced	Sheep BM-MSCs (transfected) and human dental stem cells	1 × 10^6^ (each cell type)	Light microscopy	Better bone regeneration in cell treated group animals	Feitosa et al. (2010)
Chronic model of medial femoral condyles osteochondral lesions	10 (40 defects; group I: chondrogenically differentiated MSC/hydrogel constructs; group II: undifferentiated ovine MSC/hydrogel constructs; Group III: cell free hydrogel; group IV: control	7 mm/ 6 months	Autologous BM-MSCs/collagen I hydrogel constructs	4 ×10^5^ MSCs mixed with collagen I	Histopathology	Group I had significantly better histologic scores with morphologic characteristics of hyaline cartilage such as columnarization and presence of collagen type II compared to others. However, each group showed variability in results	Zscharnack et al. (2010)
Chronic model of anterior cruciate ligament excision	16; 6 animals in group I (pre-differentiated MSCs and II (undifferentiated MSCs) and 4 (control group)	6 weeks	Chondrogenically differentiated MSCs or undifferentiated MSCs	10 × 10^6^ per joint	Gross, histological and clinical observation	Retardation of osteoarthritis in cell treated groups. Non-significant difference in group I and II except for macroscopic observations of meniscus repair. Severe osteoarthritis in control	Al Faqeh et al. (2012)
Chronic model full thickness medial femorotibial condyles and meniscal tear	10 (20 defects 10 studied at 6 months period while other 10 at 12 months period) one of the limbs remained control	60 mm defect size/6 months or 12 months	BM-MSCs	1.1 × 10^7^ (6 month period animals) or 1.2 × 10^7^ (12 month period animals)	Radiography, MRI, ultrasound, macroscopic and histological analyses	Regeneration of articular cartilage and meniscus was case-dependent but statistically significant improvement was found in specific macroscopic and histological parameters	Caminal, Moll, et al. (2014)
Medial femoro-tibial condyle defect	9 (18 defects)	7 mm defect size/4 and 12 months	BM-MSCs alone or seeded on co-polymeric poly-lactide:polyglycolic acid scaffolds either	3.3 × 10^6^±0.4 × 10^6^ cells	Biomechanical testing, macroscopic and histological analyses	Better macroscopic scores at 4 months in cell treated compared to 12 months evaluation period. Non-significant histopathological scores at 12 months between cell treated and cell free groups	Caminal, Fonseca, et al. (2014)
Chronic anterior cruciate ligament transection and medial meniscectomy	18 (6 animals in each group) Group I: BM-MSCs; group II: bone marrow mononuclear cells; group III: control	8 weeks	Autologous BM-MSCs	10 × 10^6^ after 12 weeks of model creation	Macroscopically and histologically, and glycosaminoglycan (GAG) contents, gene expression levels (collagen II, aggrecan and matrix metalloproteinase-13), tumor necrosis factor-α (TNF-α) and transforming growth factor beta	Significantly higher cartilage regeneration and lower proteoglycan loss in group I than group II. Comparable inhibition of PGE2, TNF-α and TGF-β levels in synovial fluid and promotion of higher levels of Aggrecan and Col II in two cell treated groups. Down regulation of MMP-13 also comparable. Both the cell treated groups had significantly better cartilage than control	Song et al. (2014)
Full thickness lateral femoral condyle defect	Group I (amniotic membrane); group II (cryopreserved amniotic membrane previously cultivated 12 (4 each group) with BM-MSCs; group III (cryopreserved amniotic membrane alone); group IV (control)	7 × 5 mm/8 weeks	BM-MSCs and amniotic membrane	2 × 10^6^ cells and amniotic membrane	Gross and histopathology	Significant difference between treatment and control group. Non-significant differences in treatment groups	Garcia et al. (201^4^)
Partial thickness medial femoral condyle defect	15 animals/ 30 knees (group I: scaffold plus cell; group II; scaffold only; group III control)	10 mm/6 months	Xenogenic AD-MSCs and collagen/chitosan scaffold	1 × 10^6^ along with scaffold	Microscopic and macroscopic analysis	Significantly higher histological scores in cell treated group compared to others	Zorzi et al. (2015)
Unilateral medial meniscectomy	20 (Group I: 6 animals, BM-MSCs + scaffold; Group II: 6 animals BM concentrate + scaffold; group III: 4 animals scaffold treated group IV: 4 animals, control	12 weeks	BM-MSCs + scaffold (Hyaff^®^-11) and BM concentrate + scaffold (Hyaff^®^-11)	6 × 10^6^ seeded on scaffold	Macroscopy, histology, immunohistochemistry, and micro-computed tomography	BM concentrate better inhibited inflammation in cartilage, meniscus, and synovium. It also improved cartilage healing. subchondral bone thickness decreased in both the cell treated groups	Desando et al. (2016)
Meniscal cartilage tear model	30 animals (3 groups with 10 animals in each group evaluated at 13 weeks and 6 months). Group I: scaffold laden MSCs; group II: scaffold only and group III: suturing only)	5 × 3 mm/13 weeks and 6 months	BM-MSCs collagen I scaffold	1 × 10^6^/cm^2^	Macroscopy and histopathology	Statistically significant improvement in cell treated compared to control at 13 weeks. But no difference at 6 months period	Whitehouse et al. (2017)
Anterior cruciate ligament resection and medial meniscectomy	Group I: AD-MSCs and hyaluronic acid; group II: hyaluronic acid and group III: control)	14 weeks after treatment	Allogenic AD-MSCs and Hyaluronic acid	5 × 10^7^ cells at 3 weeks) and low (1 × 10^7^ cells at 6 weeks)	Magnetic resonance imaging (MRI), macroscopy, micro-computed tomography, and cartilage-specific staining	AD-MSCs + hyaluronic acid could efficiently block osteoarthritis progression and promote cartilage regeneration.	Feng et al. (2018)

The cells were implanted either locally or by peripheral injection. Intravenous implantation of the BM mesenchymal precursor cells in a sheep mono-arthritis model leads to a reduction in their lameness, joint pain, and swelling. The joint examination revealed a decrease in the cartilage erosions, synovial stromal cell activation, and angiogenesis. Additionally, a slight infiltration of the synovial tissues with CD4+ lymphocytes and CD14+ monocytes or macrophages was observed. All these findings were contrary to those observed in the control animals (Abdalmula et al. 2017). Similar results were reported in another model of induced OA (medial meniscectomy and ACL resection) treated with an intra-articular implantation of different doses of allogenic AD-MSCs and HA 6 weeks after the surgery. The implanted cells were effectively viable up to 14 weeks and demonstrated a significant reduction in the concentration of anti-inflammatory factors (TNF‐α and IL‐6) in the joint. It was evident from the improved histological and microCT scores of the healed tissue that the progression of OA was reduced, and instead, cartilage regeneration was promoted (Feng et al. 2018).

Various comparative studies have variably supported an improvement in repair after the application of MSCs. In a comparative study on an OA model, the cartilage regenerative potential and stability of the healed tissue was found to be more in animals treated with the BM-MSCs compared to the BM mononuclear cells, the results of which were better than those observed in the control animals. Treatments using both humoral cells and chemokines inhibited PGE2, TNF-α, and TGF-β levels in the synovial fluid, and promoted an increase in the levels of aggrecan and *Col2A1* expression. Furthermore, the MMP-13 expression was downregulated in sheep chondrocytes (Song et al. 2014). Hyaluronan-laden BM concentrate containing MSCs and growth factors, upon effective implantation, prevented OA, and promoted regenerative processes in the cartilage and associated tissues. It is likely that a reduction in the inflammation resulting from both the treatments switched off the fibrotic and hypertrophic processes in the joints (Desando et al. 2016).

Variable results have been reported when an *in vivo* application of the chondrogenically differentiated MSCs produced *in vitro* was compared to that of the undifferentiated MSCs. A better healing with *in vitro* chondrogenic differentiated cells was reported in one of the studies (Zscharnack et al. 2010), while the healing in others was better but comparable (Al Faqeh et al. 2012; Bornes et al. 2018). Comparable histological scores were observed between the two groups when the hypoxia-cultured BM-MSCs (scaffold seeded and chondrogenically primed) were compared to those cultured under conditions of normoxia (scaffold seeded only) (Bornes et al. 2018). The variations may be due to differences in type of model used, cell concentrations, and the period for which follow up was conducted. Cell preservation does not have any significant deleterious effects on the cartilage healing potential of the amniotic-MSCs as comparable healing occurs upon implantation of either fresh or cryopreserved MSCs (Garcia et al. 2014).

It has been reported that, apart from the autologous or allogeneic MSCs, xenogenic AD-MSCs loaded onto the chitosan/collagen scaffold also promote cartilage healing. A higher International Cartilage Repair Society Score (ICRS-1) in animals treated with the cells compared to those of the scaffold-implanted or control animals was reported (Zorzi et al. 2015). Contrary to the above studies, one of the sheep experimental studies has failed to give any positive outcome for the OA conditions compared to the control upon intra-articular implantation of the autologous MSCs. Such observations were recorded using a 0.5 Tesla MRI system after a period of 12 weeks (Delling, Brehm, Metzger, et al. 2015). The poor response observed in this study could be due to the weak joint injury induced with meniscal damage that was incapable of inducing discernible osteoarthritic changes in the control group. Additionally, the concentration of implanted cells may not have been sufficient to address the challenge (Feng et al. 2018).

Interestingly, in all these studies, hyaline regeneration was not evident and the regenerated tissue failed to integrate with the native cartilage or subchondral bone. Additionally, the healed tissues underwent chondroid metaplasia and headed toward osteogenesis (Zscharnack et al. 2010; Caminal, Moll, et al. 2014). Moreover, the cell-treatment may lead to an improvement in the condition in the early period post-application, but later, the healing appears comparable between the treated and non-treated animals (Caminal, Fonseca, et al. 2014; Whitehouse et al. 2017). This may occur due to various reasons like continuous weight-bearing by the affected joint, inhibitory effect of inflammation on the migration and expression of MSCs, and an unclear pathophysiology of the condition. It may be concluded that the application of MSCs may benefit the treatment of OA. However, this cannot be guaranteed. Thus, further studies are required to standardize the MSC therapy for cartilage regeneration.

### 3.2. MSC studies in goats

Osteochondral studies in goats have mostly favored the use of MSCs, although it is yet to be standardized for optimal tissue regeneration (Table 2) (Murphy et al. 2003; Zhu et al. 2011; Bekkers et al. 2013; Jurgens et al. 2013; Nam et al. 2013; Pei et al. 2014; Zhang et al. 2018). In a medial femoral condyle and trochlear groove defect model, the AD-MSCs and/or stromal vascular fraction (SVF) seeded onto a collagen I/III scaffold resulted in better cartilage healing after 4 months than in animals treated with the acellular collagen I/III scaffold. Improved healing in the form of increased content of collagen type II, glycosaminoglycan, and formation of the hyaline-like cartilage was reported. The elastic modulus of the healed tissue was comparable to that of the native tissue. However, non-significant differences in the healing between the animals treated with AD-MSCs and SVF were observed (Jurgens et al. 2013). Similarly, animals treated with BM-MSCs were found to show better ICRS and O’Driscoll scores as well as cartilage-specific gene expression profiles compared to those of the control (Nam et al. 2013). In a model of medial meniscus excision and anterior cruciate ligament resection, intra-articular implantation of BM-MSCs were found to retard articular cartilage degeneration, subchondral bone sclerosis, and osteophytic remodeling at 12 weeks compared to the control group. However, severe OA was reported at later stages (Murphy et al. 2003). This may be due to the uncontrolled movement of the animals that lead to further degeneration of the joint. Combined use of tissue engineered osteochondral defect and BM-MSCs cultured in a bioreactor resulted in a better repair of the osteochondral defect compared to that of the control (graftless). Such a repair process is potentiated by mechanical stimulation of the graft (Pei et al. 2014).

**Table 2.** Chondrogenic *in vivo* preclinical experimental mesenchymal stem cell studies in goat.

Model type	Number of animals	Model defect size/study period	Biomaterial used	Cell dose	Evaluation criteria	Overall result	References
Chronic model of excision of the medial meniscus and resection of the anterior cruciate ligament	24 (6 animals cell treated/hyaluronan and 3 animals control for 12 weeks evaluation; 9 animals cell treated/hyaluronan and 6 animals for 26 weeks evaluation)	12 and 26 weeks	BM-MSCs + hyaluronan	10 × 10^6^ loaded in hyaluronan	Histochemistry	Marked regeneration of the medial meniscus. Degeneration of the articular cartilage, osteophytic remodeling, and subchondral sclerosis was reduced in cell-treated group compared to control at 12 weeks. However, later the cell treated and control had severe osteoarthritis.	Murphy et al. (2003)
Mandibular condyle Osteochondral defect model	50 (12 in each group) (group I: NELL-1-modified BMMSCs/PLGA, group II: BMMSCs/PLGA; group III: PLGA alone; group IV: control	3 mm-diameter × 5 mm-depth/6 weeks and 24 weeks	NELL-1 transfect autologous BM-MSCs and poly-lactic-co-polyglycolic acid scaffold	3 × 106 cells seeded on PLGA scaffold	Macroscopic, histology and immuno histochemistry, microCT	Group I showed Rapid and vigorous healing leading to fibrocartilage formation at 6 weeks. At 24 weeks complete repair of native articular cartilage and subchondral bone at 24 weeks. In group II: repaired completely filled the defect with fibrocartilage at 24 weeks, but the cartilage was less well-organized group I. In group III and IV the defects were poorly repaired, and no cartilage in the group IV or only small portion of cartilage in the group III was formed	Zhu et al. (2011)
Full-thickness chondral defect in medial femoral condyles	8 (16 knees) chondron and cell treatment *vs.* microfracture	5 mm/6 months	Chondron (chondrocytes in own matrix) and BM-MSCs	10% chondron/90% MSC combination at a concentration of 1× 10^6^ cells/mL	Macroscopic and microscopic scoring, biochemical analysis, histological and immunohistochemical analyses	Combination of BM-MSCs and chondrons lead to significantly better microscopic, macroscopic, and biochemical cartilage regeneration compared to microfracture treatment.	Bekkers et al. (2013)
Osteochondral defects created in medial condyles and trochlear grooves	8 (group I: scaffolds seeded with cultured AD-MSCs; group II: scaffolds seeded with SVF cells; group III: acellular scaffolds	5 × 3 mm/1 and 4 months	Stromal vascular fraction (SVF), AD-MSCs along with collagen type I/III scaffold	5 × 10^6^ (SVF) and 5 × 10^5^ (AD-MSCs) seeded on scaffold	Macroscopy, immunohistochemistry, biomechanical analysis, microCT analysis, and biochemistry	Cell treated groups had more extensive collagen type II, hyaline-like cartilage, and higher elastic moduli, and their glycosaminoglycan content in the cartilaginous layer that approached native tissue values. In control lesser regenerative effect was seen. No difference in healing was seen between SVF treated and AD-MSC treated animals	Jurgens et al. (2013)
Chronic full-thickness chondral defect in medial femoral condyles	18 (36 defects) group I: bone marrow stimulation and BM-MSCs; group II: bone marrow stimulation; group III: control	5 mm/6 months	BM-MSCs	1 × 10^7^ cells after 2 weeks after bone marrow stimulation for three consecutive weeks	Macroscopic, histology, biochemical assays (glycosaminoglycans) and gene expressions (aggrecan, collagen II and Sox9).	Hyaline-like tissue with higher glycosaminoglycans and chondrogenic gene expression in group I compared to group II that had fibrocartilage. Lowest healing in control	Nam et al. (2013)
Full-thickness femoral condyle cartilage defects	6 (microfracture and cell/scaffold groups)	6.5 mm-diameter/6 and 9 months	Human WJMSCs seeded in an acellular cartilage extracellular matrix (ACECM)-oriented scaffold	1 × 10^6^ cells seeded on ACECM	Analysis of inflammatory response, Magnetic resonance imaging, Gross morphology, Histology, Immunohistochemical and immunofluorescent staining, Biomechanical testing and Biochemical quantitative analyses	No significant differences between the two groups in immuno-inflammatory parameters. MRI demonstrated higher-quality cartilage and complete subchondral bone at defect sites in the cell treated group at 9 months. Histological revealed extracellular cartilage, cartilage lacuna and collagen type II levels were higher in cell treated group compared to the microfracture, while the cell treated group exhibited a higher elasticity modulus	Zhang et al. (2018)

In a comparative study conducted on the medial femoral condyle defect model, a combination of 10% chondron (chondrocytes in their own matrix) and BM-MSCs result in better healing than microfracture. Statistically significant microscopic, macroscopic, and biochemical cartilage regeneration was observed in the cell-treated animals compared to that observed in microfracture treatment (Bekkers et al. 2013). A comparative study on the healing of the goat mandibular condyle defect showed that the implantation of Nell-1 (growth factor that targets cells committed to the osteochondral lineage) modified the BM-MSCs/poly lactic-co-glycolic acid (PLGA) and repaired the defect by the induction of fibrocartilage at 6 weeks and with native articular cartilage by the 24th week. Implantation of undifferentiated BM-MSCs/PLGA favored the repair of the defect by fibrocartilage. The cells were found to be viable up to 6 weeks (Zhu et al. 2011). Similarly, xenogenic WJ-MSCs seeded on the ECM of the acellular cartilage lead to better cartilage and subchondral bone repair upon implantation in a femoral condyle defect model compared to that of the microfracture at the 9-month period. Better cartilage repair evidenced in the form of increased production of the ECM, lacunas and collagen type II, and higher mechanical strength (higher elastic modulus) was reported in cell/scaffold-treated animals (Zhang et al. 2018).

Unlike those of the sheep, MSCs in goats may not be able to heal the cartilage in all cases. Additionally, the continued weight-bearing on the affected joint may lead to OA, comparable in both treated as well as the control group (Murphy et al. 2003).

Therefore, the MSCs may potentially be utilized for the repair of osteochondral defects. However, the procedure is yet to be standardized and may be dependent on the cell source, its dosage, passage number of the cells, route of implantation, type of scaffold, and incorporation of the growth factor.

### 3.3. MSC studies in dogs

Unlike caprines and ovines, stem cell therapy in canines has been instituted both in preclinical experimental models (Table 3) (Mokbel et al. 2011; Yang et al. 2011; Hang et al. 2012; Yun et al. 2016; Kazemi et al. 2017) as well as in clinical cases (Table 4) (Black et al. 2008; Yoon et al. 2012; Vilar et al. 2013; Cuervo et al. 2014; Marx et al. 2014; Vilar et al. 2014; Harman et al. 2016). Barring a single study in which the cells were implanted at acupoints (bladder 54, gall bladder 29, and gall bladder 30), in all others, they have been implanted once locally (Marx et al. 2014). The cells were implanted either alone (Marx et al. 2014; Vilar et al. 2014) or with platelet-rich plasma(PRP)/fibrin (Vilar et al. 2013; Kazemi et al. 2017; Kriston-Pál et al. 2017) or hyaluronic acid (Guercio et al. 2012; Kriston-Pál et al. 2017; Li et al. 2018). The cases were followed-up for 1 month (Marx et al. 2014), 6 months (Vilar et al. 2013; Cuervo et al. 2014), 1 year (Kriston-Pál et al. 2017), and 5 years (Yoon et al. 2012).

**Table 3.** Chondrogenic *in vivo* preclinical experimental mesenchymal stem cell studies in dogs.

Model type	Number of animals	Model defect size/study period	Biomaterial used	Cell dose	Evaluation criteria	Overall result	References
Partial thickness chondral defects of lateral femoral condyle	32 (8 control; 12 group II; 12 group III)	3mm diameter and 1 mm depth/8 weeks	Autologous BM-MSCs	1.4–1.6 × 10^6^	Morphological, histological, and fluorescence analysis	Significant recovery in cell seeded groups. In group II better results at 8 weeks	Mokbel et al. (2011)
Osteochondral defects of medial femoral condyles	Group I: cell + scaffold; group II: control	4.2 mm diameter and 6 mm depth/3 and 6 months	Chondrogenically-induced BMSCs and scaffold	–	Gross morphology and by histological, biochemical, biomechanical and micro-CT analyses	Statistically significant improvement in gross and histological, and cartilage stiffness in cell/scaffold treated animals compared to control. Comparable Micro-CT analysis of the subchondral bone in two groups. Better results in later period than at early period.	Yang et al. (2011)
Osteonecrosis of the femoral head	24 (54 hip joints) group I: transgenic BM-MSCs; group II: BM-MSCs group III: control	2mm diameter and 2 mm width/12 weeks	VEGF 165 transgenic bone marrow mesenchymal stem cells or simple BM-MSCs	2 × 10^7^	Radiography, single-photon emission Computed tomography, histopathology, histomorphometric analysis and immunofluorescent staining for von Willebrand factor	Better results in group I compared to group II and group III	Hang et al. (2012)
Osteochondral defects	12 (24 (defects) group I: cell treated; group II: control	6 mm diameter and depth of 5 mm/24 weeks	Autologous BM-MSCs + platelet rich fibrin	1 × 10^6^	Macroscopic and histopathology	Group I had statistically significant improved histological features compared to control	Kazemi et al. (2017)
Chondral defects of stifle joint	24 (48 defects) group I: cell + hyaluronic treated; group II hyaluronic acid	4 mm/28 weeks	BM-MSCs + hyaluronic acid (HA)	1 × 10^7^	Macroscopy, magnetic resonance imaging (MRI), histopathology, immunohistochemistry for type II collagen	Group I had statistically significant improvement compared to group II	Li et al. (2018)

**Table 4.** Chondrogenic *in vivo* clinical mesenchymal stem cell studies in dogs.

Clinical condition/ailment	Number of animals included	Study period	Study type	Cell source	Cell dose	Evaluation criteria	Overall result	References
Chronic osteoarthritis of Coxo-femoral joint	21	90 d	Case series (Randomized, double blinded, placebo controlled trial)	Autologous AD-MSC	5× 10^6^ (20 dogs) and 4.5 × 0^6^ (1 dog)	Orthopedic examination scores, lameness and composite scores and size effect	Statistically significant improvement in cell treated as compared to placebo treated animals	Black et al. (2007)
Chronic osteoarthritis of humeroradial joint	14	180 d	Case series (Randomized, double blinded, non-placebo controlled trial)	Autologous AD-MSC	3–5 × 10^6^	Orthopedic examination Score and size effect	Statistically significant improvement in cell treated	Black et al. (2008)
Chronic osteoarthritis of humeroradial joint	4	30 d	Case series (Uncontrolled study)	Autologous AD-MSC	3–5 × 10^6^ (laden in Platelet-rich plasma or hyaluronic acid)	Clinical tests like trot pain on palpation and functional improvement in disability	Improvement with time as per owner (no statistical observation	Guercio et al. (2012)
Chronic arthritis of the hip joint	8	180		Autologous AD-MSC	15 × 10^6^	Force platform analysis	Significant improvement in cell treated cases	Vilar et al. (2013)
Hip osteoarthritis	39	180 d	Randomized Comparative clinical trial	Autologous AD-MSC *vs.* PRGF	30 × 10^6^	VAS, Bioarth Scale Assessment, Radiography, clinical exam.	Statistically significant improvement in cell treated	Cuervo et al. (2014)
Chronic arthritis of the hip joint	5	30 d	–	allogenic AD-MSC	0.2–0.8 × 10^6^	physical and orthopedic examinations	Positive outcome but no statistical data	Marx et al. (2014)
Chronic arthritis of the hip joint	10	180 d	Case control (Blinded control study)	Autologous AD-MSC	15 × 10^6^	X-ray and platform gait analysis	Statistically significant improvement in cell treated	Vilar et al. (2014)
Hip, elbow, stifle, or shoulder joints	93	60 d	Case series study (prospective, randomized, masked, and placebo-controlled)	Allogenic AD-MSCs	12 × 10^6^ (Cryopreserved with 85.1% viability)	Owner client-specific outcome measurement (CSOM) and secondary measures included veterinary pain on manipulation, veterinary global score, and owner global score	Statistically significant clinical improvement in cell treated	Harman et al. (2016)
Elbow dysplasia and osteoarthritis	30 dogs (39 elbow joints)	1 year	Case series (Uncontrolled study)	Allogenic AD-MSCs + Hyaluronic acid (0.5%)	12 × 10^6 ^ ± 3.2 × 10^6^cells	Owner and veterinarian examination, arthroscopy and histopathology	Significant improvement with hyaline regeneration	Kriston-Pál et al. (2017)
Hip, knee, radiocarpal intercarpal, elbow and ulnar osteoarthritis	10 patients	90 d/4 years (5 animals)	Case reports	Autologous AD-MSCs	3 × 10^7^	Physical examination and assessment for lameness, pain on manipulation, range of motion of the joint and functional disability	Statistically significant improvement for lameness at trot and for the range of motion of the joint. Statistically insignificant was detected for lameness at walk, pain on manipulation and functional disability	Dražilov et al. (2018)

Overall, clinical evaluation has supported the therapeutic outcome of the studies involving the following parameters: pain, visual analog scale, pain on manipulation scale, veterinary global scale, client-specific outcome measurement, quantitative force platform gait analysis, and range of motion. Histological assessment revealed that the healed tissue consisted of mixed fibrocartilage and hyaline that lacked complete integration into the native cartilage (Mokbel et al. 2011; Kazemi et al. 2017). A study in which follow-up arthroscopic evaluation was conducted revealed that the regenerated cartilage was hyaline (Kriston-Pál et al. 2017). In all these studies, the parameters for therapeutic evaluation were variable, with a lack in general consensus. Assessment of OA-associated lameness by pain assessment scales usually lack accuracy and concordance. A more advanced technique of quantitative force platform gait analysis can be used for its clinical evaluation (Vilar et al. 2014).

Various comparative studies have variably shown that repair with MSCs is better compared to that involving other available treatment options. In one of the studies, use of dog AD-MSCs gave better results at 6 months when compared with the results in which the platelet-rich growth factor (PRGF) was used (Cuervo et al. 2014). Chondrogenically induced dog BM-MSCs with a biphasic scaffold tend to show significantly improved gross and histological scores, and stiffness of the healed cartilage in comparison with that of the cell-free scaffold-implanted tissues (Yang et al. 2011). Additionally, the VEGF165 transgenesis of MSCs may further potentiate their reparative effect (Hang et al. 2012).

In addition to the factors mentioned above, the implantation period may also affect the cell-mediated effect. In one of the studies, it was demonstrated that the immediate implantation of cells after defect formation may have better outcomes than those wherein cells were implanted later (one month) (Mokbel et al. 2011). This may be due to the chronicity that may occur in the cartilage defects treated later, although further studies are required to ascertain this. One of the studies showed that the SVF also induces better healing and is comparable to that of the dog AD-MSCs implanted at acupoints (Marx et al. 2014). This could possibly be due to the additive effect of growth factors available in the latter treatment option (Kazemi et al. 2017). However, results obtained with SVF may not be recapitulated every time as its humoral/growth factor constituents are variable.

Thus, numerous factors that influence the results of stem cell therapy need to be studied. This may include application of pre-differentiated MSCs and also the cells that are transfected with the chondrogenic lineage-specific expression. Furthermore, uniform studies involving MSCs need to be conducted and their relation to sources, concentrations, inclusion of growth factors, and scaffolds need to be determined.

### 3.4. MSC studies in equines

Most equine MSC studies, whether preclinical or clinical, have failed to yield comprehensive cartilage regeneration but showed clinical improvement particularly on the basis of a reduction in the clinical symptoms and the condition of animals returning to work, as listed in Table 5 and Table 6 (Wilke et al. 2007; Frisbie et al. 2009; McIIwraith et al. 2011; Raheja et al. 2011; Spaas, Oosterlinck, et al. 2012; Yamada et al. 2013; Broeckx et al. 2014; Ferris et al. 2014; Broeckx et al. 2019). The healing appeared to be better in MSC-treated cases at the early period but decreased in the later stages (Wilke et al. 2007). Like autologous cells, single time intra-articular implantation of allogeneic BM-MSCs too has failed to elicit immune response (Ardanaz et al. 2016). However, repeated intra-articular implantation elicits adverse reactions against allogenic BM-MSCs (Joswig et al. 2017). In many such studies, an attempt has been made to mimick chronic condition of the OA by implanting cells after a gap of some days/weeks after defect creation (Wilke et al. 2007; Yamada et al. 2013).

**Table 5.** Chondrogenic *in vivo* preclinical experimental mesenchymal stem cell studies in horse.

Model type	Number of animals	Model defect size/Study period	Biomaterial used	Cell dose	Evaluation criteria	Overall result	References
Chronic Full-thickness cartilage defects in femoro-patellar articulations	6 (12 defects Group I: autogenous fibrin vehicle containing MSCs; group II: autogenous fibrin alone in control joints	15 mm/1 and 8 months	MSCs loaded into self -polymerizing autogenous fibrin vehicle	MSCs/fibrinogen mixture containing 12 × 10^6^ MSC/mL	Histology, histochemistry, collagen type I and type II, immunohistochemistry, collagen type II in situ hybridization, and matrix biochemical assays	Arthroscopic scores in group I were significantly improved at the 30-d. Biopsy showed MSC-implanted defects contained increased fibrous tissue with several defects containing predominantly type II collagen. At 8 months no significant difference between stem cell-treated and control defects.	Wilke et al. (2007)
Middle carpal joint osteochondral defect	24 8 each group (group I: AD-MSCs; group II: BM-MSC; group III: control	15 mm/70 d	AD-MSCs and BM-MSCs	16.3 × 10^6^ (AD-MSCs) and 10.5 × 10^6^ (BM-MSCs)	Clinical outcome, Macroscopy, histopathology, Articular Cartilage Matrix Evaluation	Non-significant differences in healing improvement with cell treatment were seen compared to control except improvement in PGE2 levels.	Frisbie et al. (2009)
Full thickness femoral condyle defects followed by microfracture	10 (20 defects in stifle joint) Group I: BM-MSCs and microfracture; Group II: microfracture	10 mm/6 and 12 months	MSCs and hyaluronan	20 × 10^6^ MSCs + 22 mg hyaluronan	Radiography, arthroscopy, Magnetic resonance imaging and gross, histologic, histomorphometric, immunohistochemical, and biochemical examinations	Non-significant evidence of any clinically significant improvement in the joints with BM-MSCs. Arthroscopic and gross evaluation confirmed a significant increase in repair tissue firmness and a trend for better overall repair tissue quality (cumulative score of all arthroscopic and gross grading criteria) in BM-MSC-treated joints. Immuno-histochemical analysis showed significantly greater levels of aggrecan in repair tissue treated with BM-MSC injection. There were no other significant treatment effects.	McIIwraith et al. (2011)
Chronic chondral defects in the medial femoral trochlea	4 (8 defects) group I; MSCs; group II: control	10 mm/5 months	AD-MSCs	1.35 × 10^7^	Macroscopic, histopathological and histochemical evaluations	The use of MSC in the treatment of chondral defects minimized joint inflammation, as confirmed by synovial fluid analysis. The treatment resulted in an improved repair tissue, verified by macroscopic examination, histochemical and histopathological analysis	Yamada et al. (2013)
Chondral defect, model	12 (24 defects) Group I: BM-MSCs + APEF; group II: APEF	15 mm defect in lateral trochlear ridge/3 and 12 months	BM-MSCs along with autologous platelet enhanced fibrin scaffolds (APEF)	12 × 10^6^	Arthroscopy, histological examination, magnetic resonance imaging (MRI), micro-computed tomography (micro-CT), and biomechanical testing	Addition of BM-MSCs to APEF did not enhance cartilage repair and stimulated bone formation in some cartilage defects	Goodrich et al. (2016)

**Table 6.** Chondrogenic *in vivo* clinical mesenchymal stem cell studies in horse.

Clinical condition/ailment	Number of animals	Study period	Biomaterial used	Cell dose	Evaluation criteria	Overall result	References
Bilateral articular cartilage fissure defects of the medial femoral condyles and concurrent cranial cruciate ligament injury	Single case	4 and 15 months	Autologous BM-MSCs + fibrin glue	1 × 10^7^ implanted at 90 d, 3 and 13 months period	Arthroscopic examination and performance records	At 4 months marked cartilage surface smoothing, reduction in the cartilage defect depth. Further, moderate improvement in the cranial cruciate ligament was observed. After 15 months of the initial MSC treatment the horse returned to racing and had comparable race earning to that of pre-injury records	Raheja et al. (2011)
Chronic degenerative joint disease of pastern joint	Single case	4, 8, and 12 weeks and 1 year period	Peripheral blood-derived MSCs	2.5 × 10^6^	American Association of Equine Practitioners (AAEP), radiography and pressure plate analysis	Clinical improvement observed and pressure plate analysis showed load rate (LR) symmetry ratio increased considerably in both gaits, indicating an increased speed of loading at the walk as well as at the trot. A clear improvement in peak vertical force (PVF) and vertical impulse (VI) symmetry ratios was evident at the trot, indicating an increased symmetry of the weight-bearing function of the forelimbs.	Spaas et al. (2012)
Degenerative joint disease of fetlock	20 (group I PRP; **group 2**) MSCs; group **3**) MSCs and PRP; or group **4**) chondrogenic induced MSCs and PRP	6 and 12 weeks and 6 and 12 months	Peripheral blood MSCs, platelet-rich plasma (PRP)	6.7 × 10^3^ MSCs/cm^2^ cells chondrogenically induced + 200 × 10^6^ platelets	American Association of Equine Practitioners (AAEP)	Group 4 animals generated the highest evolution scores; although it had statistically insignificant difference against group 4 in the early or late evolution score.	Broeckx et al. (2014)
Early stage fetlock degenerative joint disease	75 (50 in treated and 25 in control)	3, 6, and 18 weeks and 1 year	Chondrogenically induced Allogenic MSCs + allogenic plasma	2 × 10^6^ cells + ∼85 × 10^6^ platelets	AAEP score system	lameness scores (*p*<.001), flexion test responses (*p*<.034), and joint effusion scores (*p*<.001)	Broeckx et al. (2019)

The BM-/AD-MSCs could repair equine meniscal tear models by the production of cartilage compared to control group where the defect is either partially repaired or not at all (González-Fernández et al. 2016). In an equine femoropatellar defect model, MSCs seeded in a self-polymerizing autogenous fibrin vehicle have been used with better outcomes at early stages compared to those in which the defects in the fibrin vehicle were treated. In cell-treated animals, arthroscopic scores were significantly improved at 30 d. However, after a longer follow up period (8 months), histological scores of the cell-treated group were comparable to those of the control (Wilke et al. 2007). Hyaluronan-laden BM-MSCs, upon transplantation along with induction of microfractures in an equine induced chondral defect model, could result in better arthroscopic and gross appearances. However, an insignificant improvement in the clinical and histological examinations compared to those of the microfractures was reported after an evaluation period of 12 months. Overall, it was reported that the use of BM-MSCs led to better cartilage quality with an increased aggrecan and tissue firmness (McIIwraith et al. 2011). In another equine chondral defect model (15 mm defect in lateral trochlear ridge), it was shown that the BM-MSCs (12 × 10^6^) along with autologous platelet-enhanced fibrin scaffolds (APEF) does not carry any added advantage over APEF alone at 12-month evaluation. After 3 months, the healed tissue had a cobblestone appearance with a fair to good subchondral integration, and at the later evaluation period, the healed tissue appeared less smooth with less subchondral bone integration (Goodrich et al. 2016). Thus, it may be inferred that, in equines, the MSCs may enhance early matrix synthesis, but without any long-term benefit.

The ability of BM-MSCs in healing the cartilage tissue is considered to be better than that of the AD-SVF. Use of BM-MSCs comparably results in better clinical, biochemical, and histological improvement in osteoarthritic joints at day 70. Moreover, the animals treated with BM-MSCs are better able to reduce the PGE2 levels in the synovial fluid. Unlike BM-MSCs, AD-SVF increases the unwanted concentrations of TNF-α in the synovial fluid (Frisbie et al. 2009). Implantation of the BM aspirate in combination with the microfracture technique yields a cartilage with better macroscopic characteristics and histological and MRI scores in an equine model (15 mm full-thickness lateral trochlear ridge defects) compared to those in which the microfracture technique alone was used for an 8-month evaluation period. Although microfracture (a surgical technique to induce tiny fractures in the subchondral bone) may make the stem cells available, their cell numbers may not be sufficient enough to cause the desired amount of healing (Fortier et al. 2010). In a comparative study, implantation of MSCs along with PRP was shown to improve the functionality and sustainability of damaged fetlock joints from 6 weeks to 6 months as compared to the use of PRP alone. Implantation of chondrogenically differentiated MSCs and PRPs led to the highest short-term clinical evaluation scores right from 6 weeks through 12 weeks and from an evaluation period from 6 to 12 months (Broeckx et al. 2014). In a randomized, multicentered, double-blinded, and placebo-controlled study, intra-articular (fetlock joint) implantation of chondrogenically-induced allogenic MSCs along with the allogenic plasma has led to significant improvement in lameness scores, flexion test responses, and joint effusion scores compared to those of the control group horses. The relevant improvement to clinical therapy was seen as early as 3 weeks and continued till 18 weeks. During the various evaluation periods, significant improvement was observed in the treated cases compared to that of the control cases. However, it is worth mentioning that not all the cases displayed a same level of improvement (Broeckx et al. 2019). In contrast to sheep MSC studies, chondrogenically-differentiated equine MSCs, upon transplantation, have mostly resulted in better clinical outcomes. However, it needs to be validated whether the observed response was due to the addition of plasma or PRP to the MSCs or due to any other reason. The utility of chondrogenically-differentiated allogenic MSCs needs further validation as differentiated cells express MHC-II, and thus, may incite the immune response and get rejected.

Currently, the recommended dosage of stem cell implantation is 2 × 10^7^ in the hyaluronan scaffold (22 mg of Hyvisc (hyaluronate sodium, 3 × 10^6^ Da, Anika Therapeutics, Woburn, MA, ]) (Schnabel et al. 2013), prior to which NSAIDs are administered to reduce the joint flare (Gugjoo, Amarpal, Makhdoomi, et al. 2019). Clinical evaluation of the use of autologous BM-MSCs in 33 horses having joint affections (meniscal, cartilage, or ligamentous damage) revealed that 43% of horses returned to the previous level of work, 33% returned to work, and 24% failed to return to work (Ferris et al. 2014). In a clinical study employing a combination of PRP and chondrogenically-induced MSCs, it was found that the clinical parameters in these animals were better than those treated with PRP and undifferentiated MSCs followed by those of the PRP-treated animals (Broeckx et al. 2014). A single clinical case affected with bilateral articular cartilage fissure defects of the medial femoral condyles and concurrent cranial cruciate ligament injury has been reported, in which multiple improvements were observed by the application of BM-MSCs. The initial cell/fibrin glue mixture was delivered arthroscopically into the articular cartilage defects 90 d after the initial arthroscopic examination followed by two more cell implantations at 5 and 13 months. Evaluation by arthroscopy at 4 months (after the initial MSC treatment) revealed marked cartilage surface smoothing and a reduction in the depth of the cartilage defect. Furthermore, moderate improvement in the cranial cruciate ligament was observed. After 15 months of the initial MSC treatment, the horse returned to racing and had comparable race earning to that of the pre-injury records (Raheja et al. 2011). Similarly, in another case report, MSCs derived from the peripheral blood implanted twice at intervals of 8 weeks led to an improvement in the chronic degenerative disease of the pastern joint (Spaas, Oosterlinck, et al. 2012).

Preclinical experimental models usually provide uniform conditions to understand the effect of the MSCs. However, the results obtained therein may not be recapitulated under clinical conditions. Clinical settings, whether in animals or humans, usually provide uncontrolled studies, since variability is observed in the joint type and lesion (s) including their site and duration of existence. Additionally, age of the patient is also non-uniform. Moreover, cell sources, culture techniques, passage number, cell number, methods of implantation and addition of growth factors, and scaffolds could have a bearing on the outcome. Furthermore, incorporation of other surgical techniques and evaluation criteria need to be conducted uniformly (Gugjoo, Amarpal, Makhdoomi, et al. 2019).

## 4. Conclusions and future perspectives

Articular cartilage, once damaged, tends to undergo deterioration with each passing day due to its typical location and limited innate healing potential. With limited success of current surgical techniques, incorporation of stem cells in regenerative medical therapy is being extensively studied to enable better cartilage rehabilitation. Among the various stem cells, MSCs, particularly those obtained from adipose tissue and BM, are being studied to evaluate their clinical applications. This technology promises to develop mechanically strong cartilage-to-cartilage interface, and involves mesenchymal condensation into cellular bodies under the influence of growth factors. However, this technique is yet to be validated under *in vivo* clinical conditions. The clinical application of MSCs has mainly been adopted in dogs and horses, whereas in sheep and goats, MSCs have been mainly studied in preclinical experimental models. The chondrogenesis of MSCs highly varies with respect to the cell sources, culture techniques, passage number, number of implantations required, and incorporation of growth factors and scaffolds; thus, this warrants further studies. In general, better repair is observed upon treatment with MSCs in comparison to that of the control. However, there are various concerns associated with MSC treatment such as lack of typical hyaline tissue regeneration, integration of regenerated tissue matrix with the host native cartilage or subchondral bone, and its comparable effectiveness in all cases. Thus, further studies and experiments need to be conducted before the regenerative medicine involving stem cells can be considered fully effective and utilized clinically.
